# FOXC1 Downregulates Nanog Expression by Recruiting HDAC2 to Its Promoter in F9 Cells Treated by Retinoic Acid

**DOI:** 10.3390/ijms22052255

**Published:** 2021-02-24

**Authors:** Hongni Xue, Fayang Liu, Zhiying Ai, Jie Ke, Mengying Yu, Bingxue Chen, Zekun Guo

**Affiliations:** 1 College of Veterinary Medicine, Northwest A&F University, Yangling 712100, Shaanxi, China; xuehongni@nwafu.edu.cn (H.X.); liufayang@nwafu.edu.cn (F.L.); aizhiying@126.com (Z.A.); yb97637@um.edu.mo (J.K.); woshihaokkk@outlook.com (M.Y.); DecadeChen@outlook.com (B.C.); 2 Key Laboratory of Animal Biotechnology, Ministry of Agriculture, Northwest A&F University, Yangling 712100, Shaanxi, China

**Keywords:** F9 cells, ARTA, Nanog, promoter analysis, FOXC1 interactome, HDAC2

## Abstract

FOXC1, a transcription factor involved in cell differentiation and embryogenesis, is demonstrated to be a negative regulator of Nanog in this study. FOXC1 is up-regulated in retinoic acid-induced differentiation of F9 Embryonal Carcinoma (EC) cells; furthermore, FOXC1 specifically inhibits the core pluripotency factor Nanog by binding to the proximal promoter. Overexpression of FOXC1 in F9 or knockdown in 3T3 results in the down-regulation or up-regulation of Nanog mRNA and proteins, respectively. In order to explain the mechanism by which FOXC1 inhibits Nanog expression, we identified the co-repressor HDAC2 from the FOXC1 interactome. FOXC1 recruits HDAC2 to Nanog promoter to decrease H3K27ac enrichment, resulting in transcription inhibition of Nanog. To the best of our knowledge, this is the first report that FOXC1 is involved in the epigenetic regulation of gene expression.

## 1. Introduction

Nanog was first identified in 2003 [1,2] as a key transcription factor during preimplantation development [3]. A mouse embryo that underwent Nanog knockout was able to develop into a normal blastocyst, but failed to form an epiblast [1,4]. Expression of Nanog is essential to the stemness maintenance, reprograming, and regulation of other stem cell factors. Nanog declines along with Embryonic Stem Cells (ESCs) differentiation [5,6,7,8,9,10]. Regulation of Nanog expression is a complex and delicate process, which includes DNA and histone modification, and transcriptional activation/inhibition by other transcription factors via binding its promoter, mRNA stability and posttranslational modification [11,12,13,14,15,16,17,18]. Though it has been well-studied, Nanog regulation at the molecular level is still not completely understood. As essential factors involved in embryonic stem cells’ development, the relationship between Nanog and FOXC1 has not been studied. All-Trans Retinoic Acid (ATRA) is widely used in the study of stem cell differentiation [19,20,21,22]. Nanog is highly expressed in F9 Embryonal Carcinoma (EC) cells, which share many similarities with ESCs and are broadly used in the analysis of molecular mechanisms associated with differentiation. It is necessary to find more negative regulators of Nanog to uncover the details of its regulation during stem cell differentiation and pluripotency sustaining.

The forkhead box C1 (FOXC1) gene is located in the mouse Chromosome 13 qA3.2, containing only one exon and encoding a 553-amino-acid protein. The FOXC1 protein is a crucial transcription factor in mesoderm development [23] and is indispensable in neural crest and ocular development [24,25,26,27]. Previous studies identified two transcriptional activation domains on the N/C-terminal, one inhibitory domain located at the central region of the protein [28], and an evolutionarily conserved sequence-specific DNA binding domain known as the forkhead box domain with a nuclear localization sequence on each of its sides. Thus, FOXC1 can locate itself at the nucleus and bind to specific sites on transcriptional regulatory elements and participate in the regulation of gene expression. FOXC1 modulates gene transcription by interacting with other transcriptional factors, such as SMADs, SOX9, PITX2a, and PBX1 [29,30,31,32,33]. In recent studies, FOXC1 was considered as a pioneer factor for opening heterochromatin and regulating gene transcription [34]. However, the detailed mechanisms are still unknown, and whether FOXC1 participates in chromatin modification is also unclear. Previous studies identified FOXA1 as a negative regulator of Nanog transcription by binding to its promoter and recruiting Grg3 [35], but negative regulators of Nanog transcription were not completely revealed [36,37].

In this study, FOXC1 was screened out as a differentiation-related gene using our previous expression profile data in J1 ES cells treated with Retinoic Acid (RA) (GSE43405) [38], because it was significantly induced by RA. As an important transcription factor, we speculated that it was negatively related to sustaining pluripotency. Through the overexpression and interference of FOXC1, it was confirmed that FOXC1 can regulate the expression of Nanog. Further study showed that FOXC1 binds to the Nanog promoter and recruits its co-repressor, HDAC2, to inhibit Nanog expression by decreasing the H3K27ac level.

## 2. Results

### 2.1. Suppression of Nanog by RA Is Related to FOXC1

**Figure 1:**
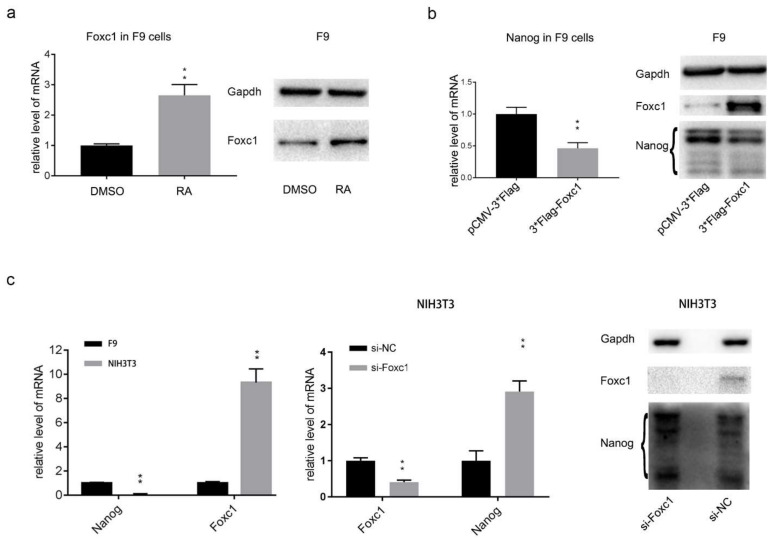
Suppression of Nanog by Retinoic Acid (RA) is FOXC1-related. (**a**) F9 cells were treated with RA for 48 h to confirm that the FOXC1 expression was increased at both the mRNA and protein levels, detected by Q-RT-PCR and Western blotting. Fold change was normalized to the mean of DMSO (±SD). (**b**) Over-expression of FOXC1 suppressed the expression of Nanog in F9 cells. Q-RT-PCR and Western blotting experiments identified that FOXC1 reduced Nanog expression at both the mRNA and protein levels. Fold change was normalized to the mean of pCMV-3*Flag (±SD). (**c**) Q-RT-PCR of FOXC1 and Nanog indicated that FOXC1 was highly expressed in NIH3T3 cells while Nanog was suppressed compared with F9 cells. Downregulation of FOXC1 reactivated Nanog expression in NIH3T3 cells at both the mRNA and protein levels. Fold change was normalized to the mean of F9 or si-NC (±SD).The statistical significance of differences was assessed by one-way ANOVA (** *p* < 0.01, * *p* < 0.05).

Activity of alkaline phosphatase in F9 cells decreased gradually after RA treatment, which significantly receded after of 48 h (Figure S1). Transcription of the core pluripotent transcription factors Oct4, Nanog, Sox2, and c-Myc decreased (GSE56893) [39]. FOXC1 was significantly up-regulated by RA treatment in J1 ESCs in our previous data, which was also confirmed in F9 cells by Western blotting and Quantitative Real-Time Polymerase Chain Reaction (Q-RT-PCR) (Figure 1a). As an important transcription factor, we speculate that the significant upregulation of FOXC1 may contribute to the downregulation of core pluripotency factors. Over-expression of FOXC1 in F9 cells suppresses Nanog expression specifically and significantly, but not other core pluripotency factors (Figure 1b and Figure S2). Due to the fact that expression levels of FOXC1 in F9 cells are too low to be interfered by si-RNA (small interfering RNA), a FOXC1 knockdown experiment was performed with NIH3T3 cells. Compared with F9 cells, FOXC1 was expressed about nine times higher in NIH3T3 cells (Figure 1c). Meanwhile, Nanog expression was hardly detectable in NIH3T3 cells (Figure 1c). Therefore, FOXC1 was knocked-down by si-RNA (si-FOXC1, si-NC) in NIH3T3 cells to find out whether Nanog will be reactivated. Total RNA and protein were collected and detected by Q-RT-PCR and WB 48 h post-transfection. As per the results shown, FOXC1 was reduced by more than half and Nanog increased by approximately four times compared to the control group at the mRNA level. The changes of FOXC1 and Nanog at protein levels were consistent with mRNA (Figure 1c). In summary, FOXC1 was found to be negatively related with Nanog expression in F9 cells.

### 2.2. FOXC1 Down-Regulates Nanog Expression by Binding Its Promoter

**Figure 2:**
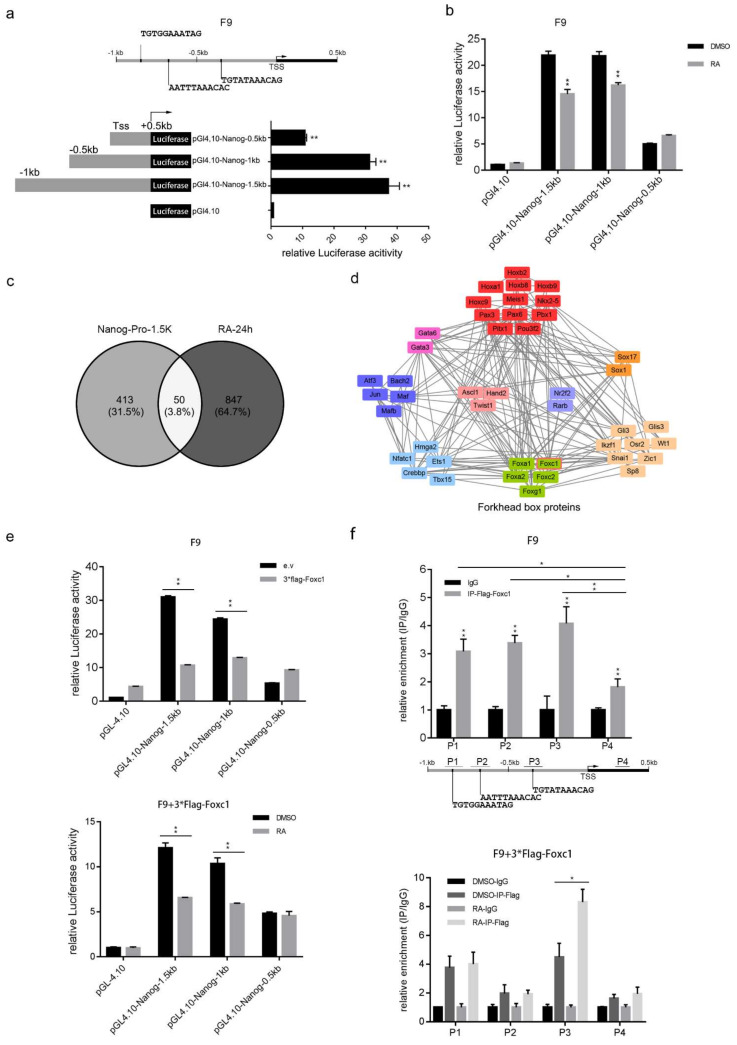
FOXC1 suppresses Nanog expression by binding to its promoter. (**a**) Three binding motifs of FOXC1 were predicted on the Nanog promoter and Double Luciferase Report (DLR) assay was carried out to detect the Nanog promoter’s activity in F9 cells. Promoter activity was normalized to the mean of the pGL4.10 empty vector (±SD). (**b**) RA suppresses Nanog promoter activity. F9 cells were transfected with promoter reporter vectors as well as treated by RA for 48 h. DLR was used for detecting the relative promoter activity, which was normalized to the mean of pGL4.10 empty vector. (**c**) Analysis of transcription factors on Nanog promoter. A total of 463 transcription factors were predicted on the Nanog promoter, and 897 genes up-regulated by RA were screened out. A total of 50 genes were found to overlap the two groups. (**d**) The 50 genes were classified using Simple Modular Architecture Research Tool (SMART) and divided into 5 groups according to their conserved domains, including the forkhead box proteins. (**e**) A DLR experiment confirmed that over-expression of FOXC1 suppressed the transcriptional activity of the Nanog promoter (the upper part) and RA increased the suppression of the Nanog promoter activity of FOXC1 (the bottom part). Values were normalized to the mean of the group: pGL4.10 + pCMV-3*Flag/pGL4.10 + pCMV-3*Flag -FOXC1-DMSO (±SD). (**f**) The FOXC1 protein located on the Nanog promoter and RA increased the enrichment of FOXC1 on P3 in F9 cells. A ChIP experiment and Q-RT-PCR identified the enrichment of FOXC1 on the Nanog promoter at P1, P2 and P3 but not on P4, which was consistent with the FOXC1 binding site prediction below the histogram (the upper part). When RA was added, FOXC1 enrichment at P3 was significantly increased (the bottom part). Fold change was normalized to the mean of IgG (±SD). The statistical significance of differences was assessed by one-way ANOVA (** *p* < 0.01, * *p* < 0.05).

Since FOXC1 has a forkhead box DNA binding domain, we speculate that it may bind to the Nanog promoter region by recognizing specific sequences. The Nanog promoter sequence (>mm10_dna range = chr6:122,706,565–122,708,065 5′pad = 0 3′pad = 0 strand = +) was submitted to the JASPAR database [40] to predict the FOXC1 binding motif and the top three sites were selected. Two of them are located at the region of 1 Kb to 0.5 Kb upstream of the Transcription Start Site (TSS), and the third one is located 0.5 Kb upstream of the TSS with the highest score (Figure 1a). The Nanog promoter of −1 Kb to +0.5 Kb relative to the TSS and its truncations was cloned to pGL4.10 luciferase report vector (Figure 2a). pGL4.10-Nanog-1.5 Kb, pGL4.10-Nanog-1 Kb, and pGL4.10-Nanog-0.5 Kb were denoted as “Nanog^1.5 Kb^”, “Nanog^1 Kb^”, and “Nanog^0.5 Kb^”. Activity of the Nanog reporter vectors were detected in F9 cells using a Double Luciferase Report (DLR) assay, and we found that the transcriptional activity of “Nanog^1.5 Kb^” and “Nanog^1 Kb^” was significantly higher compared with “Nanog^0.5 Kb^”. Deleting of 0.5 Kb upstream of the TSS severely diminished the luciferase activity of the Nanog promoter (Figure 2a). Next, an effect of RA treatment on the Nanog promoter was detected. The reporter vectors were transfected into F9 cells followed by 48 h of RA treatment. The DLR experiment showed that the transcriptional activity of “Nanog^1.5 Kb^” and “Nanog^1 Kb^” was significantly suppressed by RA treatment compared with “Nanog^0.5 Kb^” (Figure 2b). A total of 463 potential transcription factors were reversely predicted (Table S1) using the Animal TFDB3.0 online tool on the Nanog promoter region of −1 Kb to +0.5 Kb. Additionally, 897 genes up-regulated by RA treatment for 24 h in J1 ES cells were screened out (Table S2) [38,41]. Overlap of the two groups of genes was analyzed using the venny2.1 tool online and 50 transcriptional factors were identified (Figure 2c, Table 1), including FOXC1. They were clustered into five groups using SMART (Simple Modular Architecture Research Tool) (Figure 2d). In the group of Fox proteins, FOXC1 was up-regulated the highest after RA treatment. These results confirmed that in F9 cells, RA could suppress Nanog expression by down-regulating its promoter activity and that FOXC1 negatively regulates Nanog expression by binding its promoter.

We have confirmed that RA suppresses Nanog expression as well as promotes FOXC1 expression, but whether FOXC1 takes part in the suppression of Nanog promoters is unclear. Therefore, pGL4.10, “Nanog^1.5 Kb^”, “Nanog^1 Kb^”, and “Nanog^0.5 Kb^” were co-transfected with pCMV-3*Flag or pCMV-3*Flag-FOXC1. Meanwhile, the promoter reporter vectors were transfected into F9 cells followed by 48 h of RA treatment, in which FOXC1 was overexpressed. A DLR experiment was carried out after 48 h. The results showed that over-expression of FOXC1 significantly suppressed the activity of “Nanog^1.5 Kb^” and “Nanog^1 Kb^” but not “Nanog^0.5 Kb^” (the upper part of Figure 2e), and RA increased the suppression of “Nanog^1.5 Kb^” and “Nanog^1 Kb^” activity when FOXC1 was overexpressed (the bottom part of Figure 2e).

Next, to identify weather FOXC1 bound to the Nanog promoter directly, pCMV-3*Flag-FOXC1 was transfected into F9 cells. After 48 h chromatin immunoprecipitation (ChIP) combined with Q-RT-PCR experiments were carried out. In Figure 2f, primer pairs 1, 2, and 3 were designed to amplify each of the three sites, respectively, while primer pair 4 was used as a control. The fold change of Flag-FOXC1 enrichment at P1, P2 and P3 was significantly higher than that at P4. Furthermore, the effect of RA treatment on FOXC1 enrichment of the Nanog promoter was detected. RA was added to F9 cells with overexpressed 3*Flag-FOXC1 for 48 h, followed by ChIP combining Q-RT-PCR. FOXC1 enrichment at P3 was significantly up-regulated by adding RA (the bottom section of Figure 2f). In summary, FOXC1 suppressed Nanog transcription by binding its promoter region at 1 Kb upstream of the TSS and RA promoted this process by upregulating FOXC1 enrichment at −0.5 Kb relative to the TSS.

### 2.3. Identification and Analysis of FOXC1 Interactors through LC-ESI-MS/MS

To further understand the details of FOXC1 in Nanog suppression, streptavidin–biotin affinity purification combined with Liquid Chromatography Electrospray Ionization tandem Mass Spectrometry (LC-ESI-MS/MS) analysis was carried out according to our previous study [42]. Because transfection efficiency of F9 cells was too low to perform this experiment (approximately less than 30%), we tried to screen out the F9 cell line with stable expression of FOXC1 but failed because the cells could not survive. Therefore, HEK293T cells were transfected by p-Biotin and p-Biotin-FOXC1 to establish cell lines with stable expressions of Biotin and Biotin-FOXC1, named 293T-Biotin (negative control) and 293T-Biotin-FOXC1, respectively. 293T-Biotin and 293T-Biotin-FOXC1 cell lysates were precipitated by streptavidin, separated using SDS-PAGE and identified by LC-ESI-MS/MS.

**Figure 3:**
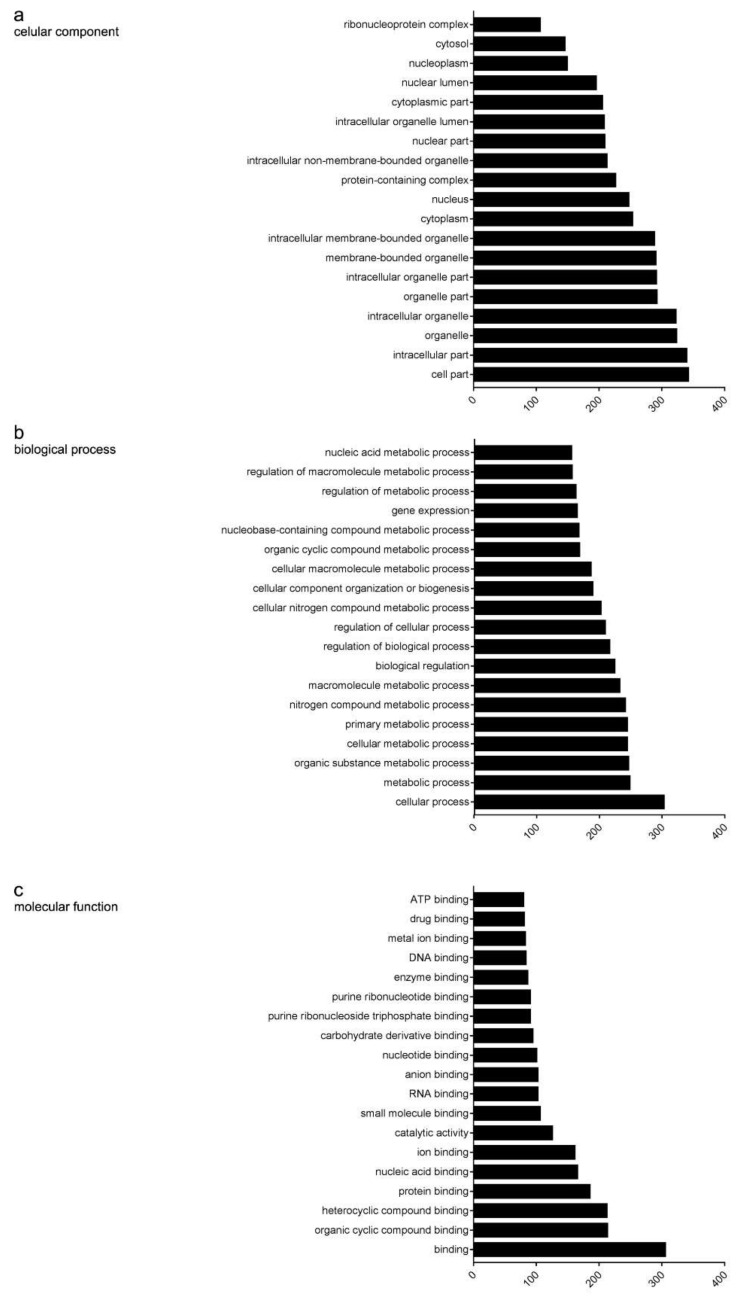
Gene Ontology (GO) classification of FOXC1 interactors. (**a**) GO biological process analysis. Cellular processes, metabolic processes and organic substance metabolic processes are the top three listed progresses. (**b**) GO cellular component analysis. Cell parts, intracellular parts and organelle are the top three listed components. (**c**) GO analysis molecular function analysis. Binding, organic cyclic compound binding and heterocyclic compound binding are the top three listed functions.

After deducting the negative control (293T-Biotin) from the results, a total of 382 proteins were identified (Table S3) and 360 of them were mapped to the STRING database (Table 2). These genes were roughly classified using Gene Ontology (GO) clustering analysis and the interaction networks were drawn using STRING [43] and Cytoscape3.4.0. As can be seen in the results, the top three items of cellular components were cell parts, intracellular parts and organelle (Figure 3a; Table S4); within the biological progress, the top-ranked categories were cellular processes, metabolic processes and organic substance metabolic processes (Figure 3b; Table S5). As for molecular function, binding, organic cyclic compound binding and heterocyclic compound binding were the major proteins (Figure 3c; Table S6). Results of a KEGG pathway analysis of the 360 FOXC1 interactors are listed in Table S7, in which the process of RNA synthesis, processing, transport, ribosome biogenesis and DNA replication and repair are included.

**Figure 4:**
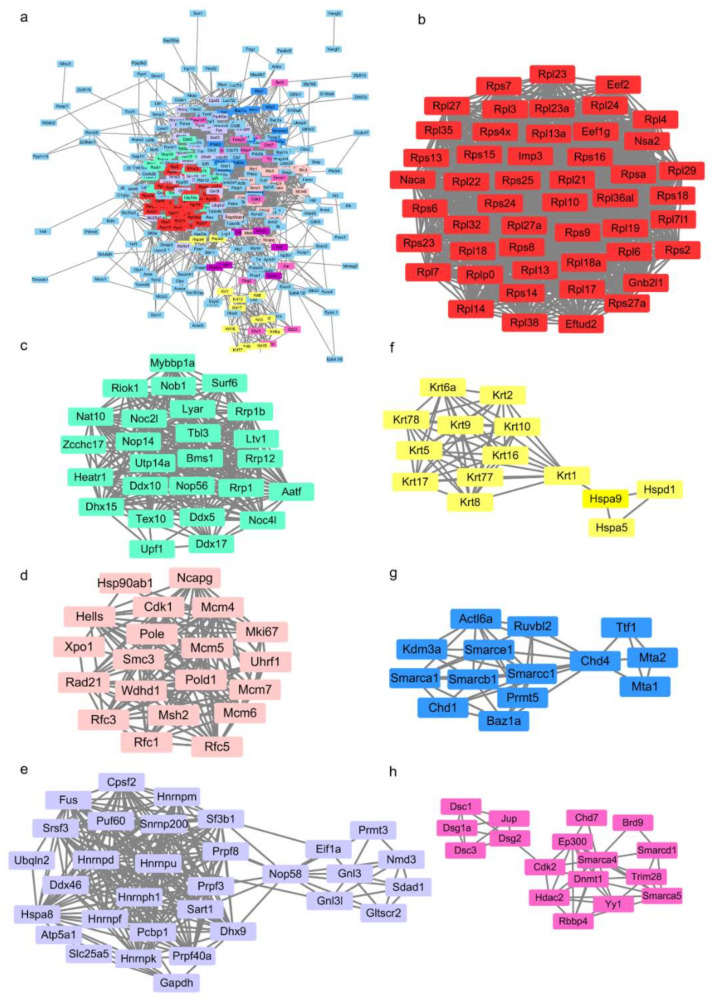
Protein interaction networks of the FOXC1 interactome. (**a**) Protein–protein interaction network of FOXC1 interactors. A total of 382 of FOXC1 interactors were submitted to the STING database and the interaction network of 360 mapped proteins was constructed with a confidence score of >0.4. The network file was downloaded and analyzed using the MCODE (Molecular Complex Detection) plugin tool in Cytoscape3.4.0 and clustered into 7 groups (with more than 10 nodes in each group). (**b**–**h**) The 7 groups of FOXC1 interactors. The protein list of each cluster was re-analyzed by STRING and classified into 5 categories: RNA synthesis, processing and transport (**b**,**c**); DNA replication, repair and metabolism (**d**); ribosome biogenesis and RNA splicing (**e**); skin development (**f**); DNA modification, chromosome remodeling and regulation of gene transcription (**g**,**h**).

To find more details about the protein interactome, the Molecular Complex Detection (MCODE) plugin tool in Cytoscape3.4.0 [44] was used to cluster the 360 mapped proteins, and seven clusters with more than 10 nodes in each group were found. According to the biological process and KEGG pathway analyses, clusters 1 and 2 are relevant to RNA synthesis, processing, and transport (Figure 4b,c); cluster 3 is related to DNA replication, repair, and metabolism (Figure 4d); cluster 4 is involved in ribosome biogenesis and RNA splicing (Figure 4e); cluster 5 is related to skin development (Figure 4f); and clusters 6 and 7 are closely related to DNA modification, chromosome remodeling and regulation of gene transcription (Figure 4g,h). These results suggest that FOXC1 performs many undiscovered functions in cell proliferation, differentiation and development by binding different partners. As shown in Figure 4c,d,h, transcription factors like LYAR and YY1 and epigenetic regulators such as UHRF1, DNMT1, HDAC2 and RBBP4 were first detected to interact with FOXC1 by LC-ESI-MS/MS. As detailed in this section, the FOXC1 interactome has been detected, but it is still unclear whether these proteins will affect Nanog expression through the interaction with FOXC1.

### 2.4. Validation of Interaction between FOXC1 with HDAC2, RBBP4, YY1, LYAR, UHRF1, and DNMT1

**Figure 5:**
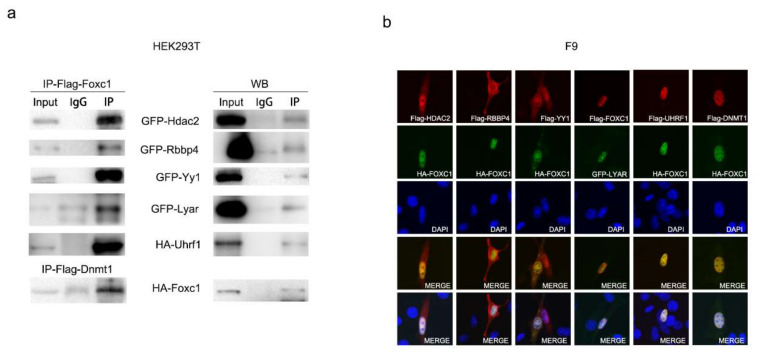
Validation of interactions of FOXC1 with HDAC2, RBBP4, YY1, LYAR, UHRF1, and DNMT1. (**a**) Co-Immunoprecipitation (Co-IP) verified that FOXC1 interacted with HDAC2, RBBP4, Yy1, LYAR, UHRF1, and DNMT1. Flag-FOXC1 and Flag-DNMT1 were immunoprecipitated by an anti-Flag antibody. GFP-HDAC2, GFP-RBBP4, GFP-Yy1, GFP-LYAR, HA-UHRF1, and HA-FOXC1 were detected using anti-GFP and anti-HA antibodies by Western blotting. (**b**) ImmunoFluorescence (IF) staining of NIH3T3 cells. Flag-tag and HA-tag were visualized using Alexa-Fluor 488 and Alexa Fluor 555, respectively. LYAR was fused with GFP-tag.

The interactions of FOXC1 with HDAC2, RBBP4, YY1, LYAR, UHRF1, and DNMT1 were validated by Co-Immunoprecipitation (Co-IP) and ImmunoFluorescence (IF) staining. PCMV-3*Flag-FOXC1 was co-transfected with pEGFP-HDAC2, pEGFP-RBBP4, pEGFP-Yy1, pEGFP-LYAR, or pCMV-HA-UHRF1 into HEK293T cells; PCMV-3*Flag-DNMT1 and pCMV-HA-FOXC1 were co-transfected into HEK293T cells. The Co-IP experiment was carried out, confirming the interaction between FOXC1 with the six proteins (Figure 5a). IF was performed in NIH3T3 cells and visualized using a confocal microscope. The co-localization of FOXC1 with HDAC2, RBBP4, YY1, LYAR, UHRF1, or DNMT1 within the nucleus was verified (Figure 5b).

Co-IP and IF confirmed the interaction of FOXC1 with the six proteins; next, we wanted to find out whether these proteins participate in FOXC1-induced Nanog downregulation.

### 2.5. FOXC1 Recruits HDAC2 to Suppress Nanog and Inhibit the Growth of F9 Cells

**Figure 6:**
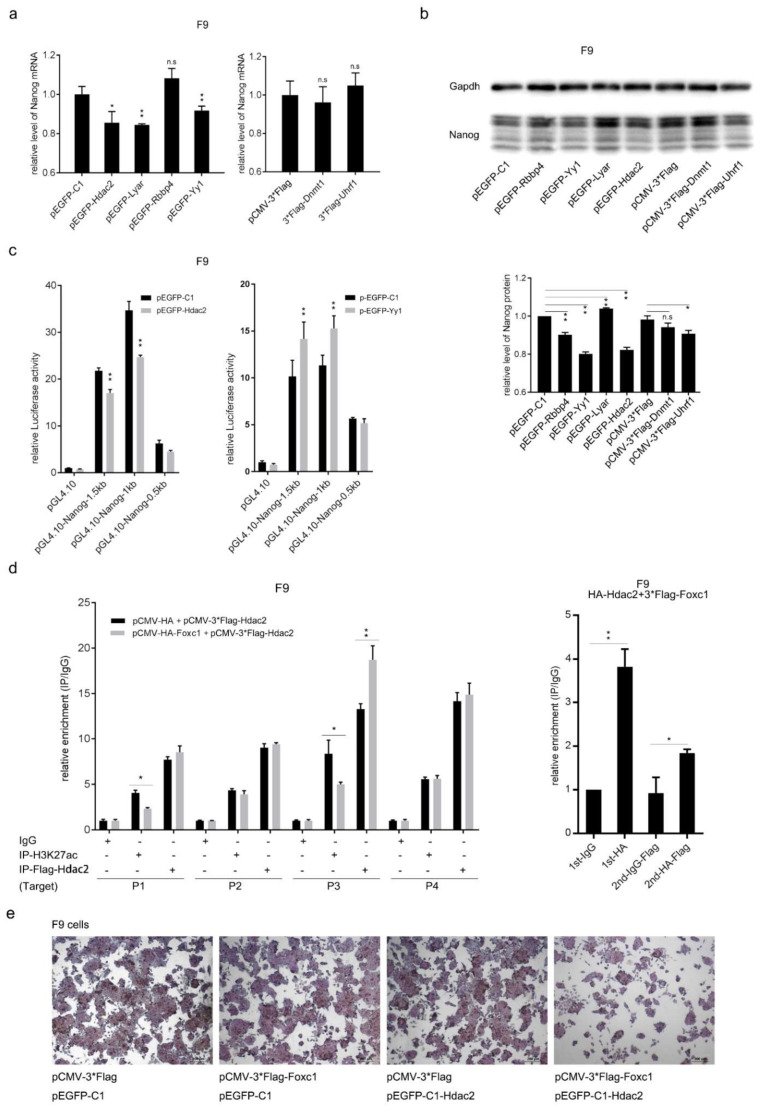
FOXC1 recruits HDAC2 to suppress Nanog and inhibit the growth of F9 cells. (**a**,**b**) HDAC2, LYAR, and Yy1 suppress Nanog transcription whereas HDAC2 and Yy1 down-regulate Nanog at both the mRNA and protein levels. Fold change was normalized to the mean of pEGFP-C1 or pCMV-3*Flag. (**c**) A DLR experiment was carried out and confirmed that GFP-HDAC2 but not Yy1decreased Nanog promoter activity. (**d**) FOXC1 promotes HDAC2 enrichment as well as reduces the H3K27ac level on the Nanog promoter at the region of P3. Flag-HDAC2 was overexpressed with or without HA-FOXC1 in F9 cells; ChIP and Q-RT-PCR experiments were performed 48 h later (**right**). HA-FOXC1 and 3*Flag-HDAC2 were co-overexpressed in F9 cells. ChIP-reChIP combining Q-RT-PCR was used to detect the enrichment of HA-FOXC1 (the first round of ChIP) and Flag-HDAC2 (the second round of ChIP) at the region of P3 on the Nanog promoter. Fold-change was normalized to the mean of IgG (±SD). (**e**) Alkaline phosphatase staining of F9 cells after over-expression of Flag-FOXC1 and GFP-HDAC2. The statistical significance of differences was assessed by one-way ANOVA (** *p* < 0.01, * *p* < 0.05).

The effects of these six factors on Nanog expression and promoter activity were detected. First, they were overexpressed in F9 cells (pEGFP-C1 and pCMV-3*Flag were the negative controls). After 48 h, total protein and mRNA were harvested and detected by Western blotting and Q-RT-PCR. The results suggest that HDAC2, LYAR, and Yy1 down-regulate Nanog at the mRNA level (Figure 6a) and that Yy1 and HDAC2 suppressed Nanog at both the protein and mRNA levels (Figure 6a,b). Next, the effect of HDAC2 and Yy1 on Nanog promoters were tested using DLR. PEGFP-HDAC2, or PEGFP-Yy1 were co-transfected into F9 cells with pGL4.10, “Nanog^1.5 Kb^”, “Nanog^1 Kb^”, and “Nanog^0.5 Kb^”, respectively. After 48 h, the DLR experiment was implemented and the result showed that HDAC2 suppressed the activity of the Nanog promoter region of 1 Kb upstream of the TSS, whereas Yy1 increased the activity of the Nanog promoter (Figure 6c).

H3K27ac is the marker of activated promoters, which could be deacetylated by HDAC2 to suppress gene transcription [45]. In ES cells, H3K27ac was highly enriched on the Nanog promoter (Figure S3) [46]. It is necessary to figure out whether HDAC2 participates in suppressing Nanog by reducing the H3K27ac level. PCMV-3*Flag-HDAC2 was co-transfected with pCMV-HA-FOXC1 into F9 cells. After 48 h, a ChIP experiment was carried out using anti-Flag and anti-H3K27ac antibodies (IgG was the negative control). The enriched DNA was detected by Q-RT-PCR. Flag-HDAC2 and H3K27ac were enriched at the fragments of P1, P2, P3, and P4. Flag-HDAC2 was enriched approximately 13 times at P3. The enrichment of H3K27ac at P3 and P4 was higher than that at P1 and P2. When FOXC1 was added, HDAC2 enrichment at the region of P3 was increased but not the other three fragments. Meanwhile, the H3K27ac level on P3 was significantly reduced (Figure 6d, left).

Furthermore, ChIP-reChIP was performed to confirm the interaction of FOXC1 and HDAC2 on the Nanog promoter. HA-FOXC1 and 3*Flag-HDAC2 were co-overexpressed in F9 cells. After 48 h, anti-HA antibodies or IgG were used to perform the first round of immunoprecipitation (IP), after which the immunocomplexes were eluted from the beads and used for the second round of IP using anti-Flag antibody. Four groups of DNA fragments were extracted as the standard procedure ChIP experiment: 1st-IgG-DNA, 1st-HA-DNA, 2nd-IgG-Flag-DNA, and 2nd-HA-Flag-DNA. Q-RT-PCR was used to determine the fold change of enrichment. The first round of IP by HA antibody caused an approximately four-fold enrichment of FOXC1 compared with 1st-IgG-DNA. The second round of IP by Flag antibody detected an about two-fold significant enrichment of Flag-HDAC2 compared with 2nd-IgG-Flag-DNA (Figure 6d, right). Therefore, the interaction of the two proteins at the same position was proven. These results suggest that FOXC1 suppresses Nanog expression by interacting and recruiting HDAC2, which reduces H3K27ac level at P3 within the Nanog promoter region of 0.5 Kb upstream of the TSS.

FOXC1 was identified as a negative regulator of Nanog expression when F9 cells were treated with RA, and its interacting protein, HDAC2, was recruited as a co-repressor to decreased H3K27ac on the Nanog promoter. PCMV-3*Flag + pEGFP-C1, PCMV-3*Flag-FOXC1 + pEGFP-C1, PCMV-3*Flag + pEGFP-HDAC2, and PCMV-3*Flag-FOXC1 + pEGFP-HDAC2 were co-transfected into F9 cells, and 48 h later, Alkaline phosphatase staining was implanted, and we found that co-expression of FOXC1 and HDAC2 significantly inhibited the growth and slightly decreased the activity of alkaline phosphatase of F9 cells (Figure 6e).

## 3. Discussion

F9 cells were derived from mouse testicular teratoma by implanting a six-day-old embryo into a 129J mouse [47], which was widely used in analysis of molecular mechanisms associated with differentiation because it has the ability to differentiate to all three germ layers in vitro. It also shares similar mechanisms in the regulation of gene expression, signaling pathways and gene expression profile (GSM1234312, and GSM757807) compared with ESCs [48,49,50,51,52,53,54]. F9 cells can differentiate into endodermal-like cells via RA treatment [49], meanwhile RA treatment caused the core pluripotency factors, such as Nanog, Sox2, and oct4, to down-regulate [55]. This similarity enables the current study to be practicable in F9 cells.

Pluripotency is the characteristic of ESCs enabling them to generate various types of cell lineages of developing and adult organism. Naïve epiblast cells, a population of unrestricted pluripotent cells, can be immortalized in culture in the form of ESCs [56,57,58]. Nanog is an essential factor to induce and safeguard pluripotency [59,60]. RA plays multiple roles in cell development and differentiation. It causes cell cycle arrest and apoptosis and inhibits cell growth, angiogenesis, and metastasis in various ways [61]. Over the past years, researchers deepened the studies on gene expression regulation at epigenetic levels, which include many kinds of histone, DNA, and RNA modifications [62,63,64,65]. Previous studies identified that Nanog expression was affected by the enrichment of H3K4me3/H3K27me3 on its promoter caused by EZH2, and methylation of its promoter caused by recruitment of MBD2 [66,67]. Our study improved the understanding of epigenetic mechanisms of Nanog suppression by FOXC1 and HDAC2 in F9 cells treated by RA.

Fox proteins include 50 and 44 members in humans and mice, respectively, which are divided into 19 subfamilies. They all have a conserved DNA binding domain named forkhead domain [68]. Fox family proteins were proven to be essential transcription factors for developmental processes such as the establishment of the body axis and the development of all three germ layers, including FOXC1 [69]. However, there have been few studies of FOXC1 as related to stem cell differentiation. Functions of FOXC1 are still controversial and ambiguous to this day and it seems that FOXC1 plays multiple roles in different cell lines by binding with virous co-factors. In this study, we demonstrated for the first time that FOXC1 interacts with the epigenetic regulator HDAC2 to affect histone post-translational modification and regulate gene expression. FOXC1 also has the potential to recruit other epigenetic regulators, such as DNMT1 and UHRF1, to affect the methylation of DNA and H3K9 during stem cell differentiation, which requires further research and more evidence in ESCs.

The protein interactome of FOXC1 was detected to uncover more details in Nanog regulation, which could also provide critical information for the functional study of FOXC1 in the future. There are only 52 interactors of FOXC1 in BioGRID (Biological General Repository for Interaction Datasets) (Table S9). After analysis of the interactors using STRING and Cytoscape3.4.0, only three incompact clusters with low scores were generated (data not shown), from which we could not obtain enough information to analyze and predict the functions and biological processes in connection with FOXC1. FOXC1 and its interactors were co-immunoprecipitated using the streptavidin–biotin affinity purification technique combined with LC-ESI-MS/MS in HEK293T cells. After deducting the negative control, 382 interactors were detected and submitted to the STRING database and 360 of them were mapped. UniProt keywords summarized the content of a protein and constituted a controlled vocabulary with a hierarchical structure. These 360 proteins were analyzed using STRING online and 63 UniProt keyword items were enriched. The 52 proteins from BioGrid were enriched in 20 UniProt keyword items. A total of 13 items overlapped between the two groups, which indicated that our data was largely consistent with the online data. Huang proved that FOXC1 interacts with human P32, which is an interactor of splicing factor of ASF/SF2 and CDC2L5. P32 was also proven to be related to transcription reaction [70,71,72,73]. Zhu [32] identified that FOXC1 interacts with PBX1 to regulate ZEB expression. Bin [74] identified FOXC1 as a regulator specific for keratinocyte terminal differentiation and established its potential position in the genetic regulatory network. In our data, a large number of unknow interactors of FOXC1 were detected, but more detailed functions in DNA replication, repair, and modification and chromosome remodeling are still unknown. These results will provide large amounts of information for FOXC1 functional study in modulating gene expression in the future.

In this study, the LC-ESI-MS/MS experiment was performed in HEK293T cells but not F9 or NIH3T3 cells. At first, we tried this in F9 and NIH3T3 cells but could not get stably transfected cells. In our previous study, HEK293T cells were used to analyze PGC7-interacting proteins [42]. Thus, we chose HEK293T to perform the LC-ESI-MS/MS experiment. It is a fact that protein interactomes are cell-specific and we will make more efforts to define the interactors detected in this study using pluripotent cells in our further research.

FOXC1 affects gene expression by binding their promoters or cooperating with other factors, and it also participates in signal transduction by working as a substrate of ERK1/2 to be phosphorylated at S272, which could increase its stability by reducing the proteasomal degradation of FOXC1 [75,76,77]. Though Functions of FOXC1 in signal transduction have been well-studied [78,79,80,81], our data suggest that FOXC1 may play unrevealed roles in the epigenetic regulation of gene expression. As seen in Figure 4h, the proteins DNMT1, HDAC2, RBBP4, Yy1, EP300, Trim28, and Smarca1/4/5 are involved in chromatin modification, regulation of chromosomal remodeling and gene expression. As seen in Figure 4b, LYAR was identified to mediate the recruitment of Brd2 to antagonistic Nanog downregulation via RA treatment [82]; and as seen in Figure 4c, UHRF1, an epigenetic integrator, recruits DNMT1 and mediates cross-talk between H3K9 methylation and DNA methylation [83]. It also participates in heterochromatin formation by interacting with G9a, Trim28, and HDACs [84,85]. The HDAC co-suppressor complexes play multiple roles in the epigenetic regulation of gene expression through remodeling chromatin and modifying other transcription factors [86]. DNMT1 is essential in epigenetic reprogramming during early embryogenesis because it is capable of methylating hemi-methylated DNA and coordinating with other factors to affect target genes [87,88,89]. RBBP4 and Smarca1/4/5 are generally accepted as chromatin remodeling factors to regulate gene expression [90,91,92,93,94]. As a result of this study, we have verified that FOXC1 interacts directly with HDAC2, RBBP4, Yy1, LYAR, DNMT1, and UHRF1. The interaction between FOXC1 and these chromatin modifiers suggested that FOXC1 may take part in epigenetic regulation, but the specific mechanisms need to be determined.

In this study, HDAC2 was first verified to be an interacting protein of FOXC1. Our results identified that the Nanog promoter region of −1 Kb to +0.5 Kb relative to the TSS is transcriptionally activated and the region of 0.5 Kb upstream of the TSS is critical for Nanog promoter activity and its response to RA. Similarly, deleting this region also negated the response of the Nanog promoter to FOXC1. By adding RA to the culture medium, suppression of Nanog by FOXC1 was intensified. The results above suggest that the region of 0.5 Kb upstream of the TSS is crucial in Nanog transcription regulation relative to FOXC1 and RA. Furthermore, the ChIP experiment confirmed that HDAC2 interacts with FOXC1 directly and is recruited by FOXC1 to the Nanog promoter region of 0.5 Kb upstream of the TSS to reduce the H3K27ac level at P3. FOXC1 enrichment at P3 was also upregulated by RA treatment. The enrichment of H3K27ac at the promoter region correlates with the nearby gene transcription [95]. All these results prove that the Nanog promoter region of 0.5 Kb upstream of the TSS is crucial to the regulation of Nanog expression by FOXC1 and HDAC2.

In summary, our results illustrate that, in the process of RA-induced Nanog suppression, FOXC1 promotes HDAC2 recruitment on the Nanog promoter, which results in the deacetylation of H3K27ac and the inhibition of Nanog transcription. This study provided a large amount of information about biological processes that FOXC1 may be involved in. For further research, the validation of more interaction proteins in ESCs warrants in-depth inquiry. Since many epigenetic regulators were detected to interact with FOXC1, they might affect gene expression together through modulating epigenetic markers, such as post-translational modification of histones and DNA methylation. Epigenetic regulation plays important roles in stem cell differentiation, pluripotency sustaining and inducing of pluripotent cells. In addition, suppression of FOXC1 might promote induced Pluripotent Stem Cells (iPSCs) because interfering of FOXC1 in NIH3T3 cells reactivates Nanog expression, which is the dispensable factor in inducing pluripotent cells [96].

## 4. Method

### 4.1. Cell Culture, Transfection, and Drug Treatment

F9 or HEK293T cells were cultured in DMEM (Dulbecco’s Modified Eagle’s Medium) (Gibco, #12800–082, Waltham, MA, USA) culture medium containing 10% FBS (Fetal Bovine Serum) (BI, #04-002-1A, Israel), maintained at 37 °C and 5% CO_2_ in a humidified incubator (Thermo Fisher, Waltham, MA, USA). Lipofectamine™ 2000 Transfection Reagent (Thermo Fisher, #11668019) was used for transfection experiments following the manufacturer’s instructions. Transfection conditions were optimized for F9 cells by using a plasmid-to-transfection reagent ratio of 1 μg: 3.5 μL. RA was added into culture medium at the final concentration of 1 uM (stock solution: 10 mM dissolved in DMSO) and an equal amount of DMSO was added to the NC groups.

### 4.2. Reverse Transcription and Q-RT (Quantitative Real Time)-PCR

Cells were collected and total RNA was extracted using RNAiso Plus (Takara, #9109, Dlian, China) chloroform extraction as well as isopropanol precipitation, and then reverse-transcribed for Q-RT-PCR using the Prime-Script RT reagent kit (Takara, #RR037A). Q-RT-PCR was carried out according to the instructions of TB Green Premix Ex Taq II (Takara, #RR820A). Primer pairs of specific genes are listed in Table S8; Actb (β-Actin) was used as the internal control.

### 4.3. Construction of Eukaryotic Expression Vectors

The sequence of target genes was downloaded from NCBI (NM_ 008592.2) and primer pairs were designed using Primer Premier version 5.0 (http://www.premierbiosoft.com/primerdesign/, accessed on 15 June 2020, San Francisco, CA, USA). The total RNA of F9 or 3T3 cells was reverse-transcribed using the PrimeScript™ II 1st Strand cDNA Synthesis Kit (Takara, #6210A). Genes were cloned and purified using PrimeSTAR^®^ HS DNA Polymerase (Takara, #R010A) and StarPrep Gel Extraction Kit (GenStar, #D205-01, Beijing, China), and then double-digested by restriction enzymes (NEB: Xho1, #R0146S; BamH1, #R3136S; EcoR1, #R3101S, Ipswich, MA, USA). PCMV-3*Flag was also double-digested and ligated with the gene segment using T4 DNA Ligase (Takara, #2011A).

Genomic DNA of F9 cells was extracted using TIANamp Genomic DNA Kit (TIANGEN, #DP304-03, Beijing, China) and Nanog promoter fragments (1.5k, 1k, 0.5k) were cloned to pGL4.10. Primer pairs are listed in Table S8.

Endotoxin-free plasmids were extracted using the Endo-Free Plasmid Mini Kit II (OMEGA, #D6950, Norcross, GA, USA) according to the instructions.

### 4.4. Western Blotting

Cells were collected in 1.5 mL tubes and centrifuged at 1000 rpm for 5 min and resuspended with the correct amount of PBS, after which 5×SDS-loading-buffer (pH 6.8 250 mM Tris-HCl, 100 g/L SDS, 5 g/L bromophenol blue, 500 g/L glycerol, 50 mL/L β-mercaptoethanol) was added into the cell suspension with the ratio of 1:4. Then the samples were put into boiling water with a vortex till the samples were not viscous. After that, the samples were centrifuged at 4 °C and 12,000× *g* for 10 min to discard the precipitation, and the supernatants were stored at −80 °C.

Protein samples were separated using SDS-PGAG (Sodium Dodecyl Sulfate Poly-Acrylamide Gel Electrophoresis) at 80 v, at constant voltage, for 2 h in a running buffer (3.03 g/L Tris, 18.8 g/L glycine, 1 g/L SDS) and transferred to a methanol pre-soaked Polyvinylidene Fluoride (PVDF) membrane (Millipore, #ISEQ00010, Burlington, MA, USA), at 250 mA, constant current, for 3 h in a transfer buffer (3.03 g/L Tris, 14.4 g/L glycine, 200 mL/L methanol). Next, membranes were blocked in 10% skim milk powder dissolved in TBST (8.8 g/L NaCl, 2.423 g/L Tris, 0.5 mL/L Tween-20; pH 7.4) and specific primary and Horseradish Peroxidase (HRP)-linked secondary antibodies were diluted with 5% skim milk powder dissolved in TBST followed by incubation at 4 °C overnight. Signal detection and visualization were completed using the WesternBright ECL kit (Advansta, #K-12045-D50, Menlo Park, CA, USA) and Gel Doc™ XR+ and ChemiDoc™ XRS + Systems with Image Lab™ Software, version 5.2.1 (BIO-RAD, Hercules, CA, USA).

### 4.5. Immuno Fluorescence and Alkaline Phosphatase Staining

In NIH3T3 cells, PCMV-HA-FOXC1 was co-transfected with pCMV-3*Flag-HDAC2, pCMV-3*Flag-RBBP4, pCMV-3*Flag-Yy1, pCMV-3*Flag-UHRF1, and pCMV-3*Flag-DNMT1; PCMV-3*Flag-FOXC1 and pEGFP-LYAR were also co-transfected into NIH3T3 cells. The culture medium was discarded and the cells were washed three times using Phosphate-Buffered Saline (PBS). Then the cells were fixed and permeabilized with Immunol Staining Fix Solution (Beyotime, #P0098, Shanghai, China) for 15 min. Blocking Buffer for Immunol Staining (Beyotime, #P0260) was used for blocking the cells (15 min at room temperature). The primary antibodies (Monoclonal ANTI-FLAG^®^ M2 antibody produced in mouse and rabbit HA Tag Rabbit Polyclonal Antibody) were dissolved in primary antibody dilution buffer for immunol staining at the ratios of 1:500 and 1:100, respectively (Sigma Aldrich, #F1804, Saint Louis, MO, USA; Beyotime, # AF0039; Beyotime, #P0262). The cells were incubated with the primary antibodies overnight at 4 °C and then incubated with the secondary antibodies. Alexa Fluor 488 and Alexa Fluor 555 were dissolved in secondary antibody dilution buffer for immunofluorescence at the ratio of 1:500 (Thermo Fisher Scientific, #A-11034, #A-21422; Beyotime, #P0265). DAPI (2-(4-Amidinophenyl)-6-indolecarbamidine dihydrochloride) staining was performed at room temperature for 10 min before visualization (Beyotime, #C1002). After each step, the cells were washed three times for 5 min using PBS with 0.1% TritonX-100 (Sangon, #A600198, Shanghai, China). The images were captured using a confocal microscope (Olympus, FV1000MPE, Tokyo, Japan).

F9 cells were treated or transfected as required, and Alkaline Phosphatase staining was performed following the instructions of the BCIP/NBT Alkaline Phosphatase Color Development Kit (Beyotime, #3206).

### 4.6. Double Luciferase Report Assay

Expression vectors and promoter reporter plasmids were co-transfected with pGL4.73 (100:10:1) into F9 cells and 48 h later, a lysis buffer was added into wells and incubated for 20 min with a shake rate of 180 rpm. Relative luciferase activity was detected using the Double-Luciferase Reporter Assay Kit (Transgen, FR201-02, Beijing, China) and the VICTOR X5 multilabel plate reader (PerkinElmer) and normalized to Renilla luciferase.

### 4.7. LC-ESI-MS/MS Analysis

BAD and BirA nucleotide sequences were cloned from the pCDH-Kozak-Flag-BAD-MCS-P2A-Myc-BirA-T2A-puro-MSCV preserved in our laboratory and ligated to pCDH-MCS-T2A-Puro-MSCV (System Biosciences, Palo Alto, CA, USA), named pBiotin (pCDH-Kozak-Flag-BAD-MCS-P2A-Myc-BirA-T2A-Puro-MSCV). FOXC1 was cloned from pCMV-3*Flag-FOXC1 and constructed to pBiotin at the Multiple Cloning Site (MCS), named pBiotin-Flag-FOXC1. Primer pairs are listed in Table S8.

PBiotin-Flag-FOXC1 and pBiotin empty vectors were co-transfected into HEK293T cells, with lentivirus packaging vectors pMD2.G and psPAX, respectively. After 48 h, supernatants of the culture medium containing the virus were collected by centrifuging at 4 °C and 5000 rpm for 10 min. When the HEK293T cells achieved about 30% confluency, the viral supernatants and puromycin (2 μg/mL) were added to a 60 mm petri dish to screen stably transfected cells.

Sufficient amounts of stably transfected HET293T cells (293-Biotin and 293-Biotin-FOXC1) were collected and lysed by nuclear extract buffer A (20 mM HEPES, pH 7.9; 10 mM KCl; 1 mM EDTA; 0.1 mM NaVO\textsubscript{4}; 0.2% NP40; 10% glycerin; 0.5 μM DTT; 0.5 μM PMSF) on ice for 10 min, after which cell nuclei were collected by centrifugation at 5000× *g* and 4 °C and lysed by nuclear extract buffer B (20 mM HEPES pH 7.9; 10 mM KCl; 1 mM EDTA; 0.1 mM NaVO_4_; 350 mM NaCl; 20% glycerin; 0.5 μM DTT; 0.5 μM PMSF) for 30 min on ice with mixing by inversion. The lysates were centrifuged at 17,000× *g* for 10 min at 4 °C, the supernatants were transferred to new centrifuge tubes and the protein concentration was determined using the BCA Protein Assay Kit (Takara, T9300A). IP350 buffer (350 mM NaCl; 20 mM Tris-HCl, pH 7.5; 0.5% NP40; 1 mM EDTA; 10% glycerin) was used for diluting the protein samples to a final concentration of 2 mg/L and 100 μL of streptavidin–agarose per 10 mg of protein was added into the samples (293-Biotin and 293-Biotin-FOXC1) to enrich the protein complexes. The procedures of washing, elution, SDS-PAGE, in-gel digestion and LC-ESI-MS/MS analysis based on Triple TOF 5600 were described in a previous article (experimental procedures are described in File S1, and the mass spectrometry proteomics data have been deposited to the ProteomeXchange Consortium via the PRIDE partner repository with the dataset identifier PXD022178) [42,97].

A total of 382 differential protein IDs were screened out by comparing the data of the background group and the 293-Biotin-FOXC1. By searching the UNIPROT database and submitted to the STRING database, a total of 360 protein IDs were mapped. The files of interaction network information, GO cluster (MF, Molecular Function; CC, Cellular Component; BP, Biological Process) and KEGG pathway analysis of the 360 proteins were downloaded from STRING. The interaction network file was analyzed using the MCODE plugin tool in Cytoscape3.4.0 (default parameters: degree cutoff: 2; node score cutoff: 0.2; K-core: 2; max. depth from seed: 100) [42,44]. Seven clusters, with more than 10 nodes in each cluster, were generated and these groups of proteins were also submitted to the STRING database to be analyzed; the files of the GO clusters and KEGG pathway were downloaded to obtain more detailed functions for each group.

### 4.8. Co-IP (Co-Immunoprecipitation)

pCMV-3*Flag-FOXC1 was transfected into HEK293T cells using Lipofectamine 2000 and 48 h later, cells were collected by centrifuge at 1000 rpm for 5 min and lysed by Pierce IP Lysis Buffer (Thermo Fisher, #87788; Protease Inhibitor Cocktail 3, #P8340; Sigma Aldrich, #P0044) on ice with vortex blending every 5 min. The cell lysate was centrifuged at 12,000× *g* at 4 °C for 10 min to be collected and divide the supernatant equally, before which 10% of the total volume of the supernatant (input) was transferred to another tube used as the positive control. An equal amount of lysate was incubated with IgG (Beyotime, #A7028) or anti-Flag antibodies (Sigma Aldrich, #F1804) for over 10 h at 4 °C and then 20 μL protein A/G agarose (Thermo Fisher, #02422) was added to precipitated the antibody–protein complexes by incubating for 2 h at 4 °C with rotation. Agarose beads–antibody–protein complexes were collected by centrifuging at 2500 rpm for 5 min at 4 °C and washed three times using IP Lysis/Wash Buffer (25 mM Tris-HCl, pH 7.4, 150 mM NaCl, 1% NP-40, 1 mM EDTA, 5% glycerol) while rotating at 4 °C, and 60 μL of 1×SDS loading buffer was added into the precipitations to denature the protein samples by boiling at 100 °C for 10 min.

### 4.9. ChIP (ChIP) and ChIP-reChIP

ChIP experiments were carried out according to the instructions of the of Pierce™ Magnetic ChIP Kit manufacturer (Thermo Fisher, #26157). Briefly, F9 cells were transfected with pCMV-Flag-FOXC1 and, after 48 h, fixed using 1% formaldehyde at room temperature for 10 min, followed by adding glycine (final concentration, 125 mM) for terminate crosslinking. Cells were scraped and transferred into centrifuge tubes and collected by centrifuging at 3000× *g* at 4 °C for 5 min, after which the cell pellets were resuspended and lysed by adding 200 μL of Membrane Extraction Buffer (MEB) containing protease/phosphatase inhibitors, vortex blending for 15 s and incubating on ice for 15 min. Nuclei were collected by centrifuging at 9000× *g* for 5 min at 4 °C and resuspended in MNase (Micrococcal Nuclease) digestion buffer working solution, and chromatin was digested to about 150–1000 bp DNA fragments using MNase in optimized conditions (4U of MNase used for approximately 4 × 10^6^ cells, digesting at 37 °C for 18 min). Digestion was terminated by adding EDTA and nuclei were collected by centrifuging at 9000× *g*, 4 °C for 5 min. Then, nuclei were resuspended in IP dilution buffer and sonicated on ice to break the nuclear membrane. The supernatants were centrifuged at 9000× *g* for 5 min at 4 °C and transferred to a new tube of which 2% of its volume was used as input (positive control) and the rest was divided equally, incubating with IgG, anti-Flag and anti-H3K27ac antibodies (Sigma Aldrich, #F1804; CST, #8173, Massachusetts, USA) at 4 °C for 10 h. A total of 20 μL of magnetic beads were added to each IP, which were incubated at 4 °C overnight with rotation. Beads–antibody–protein–DNA complexes were collected and washed three times followed by decrosslinking, protease K digestion and DNA recovery using a DNA Clean-up column. DNA samples of the input and IP were diluted at the ratios of 1:100 and 1:10, respectively, and amplified by Q-RT-PCR to determine the enrichment at specific sites.

ChIP-reChIP was performed according to a previous study [97]. HA-FOXC1 and 3*Flag-HDAC2 were co-overexpressed in F9 cells for 48 h. The first round of ChIP was performed using anti-HA antibodies (CST, #3724T), and the second round of ChIP was performed using anti-Flag antibodies.

Primers are listed in Table S8.

## Figures and Tables

**Table 1:** 50 shared genes of 897 up-regulated genes by RA treatment and 463 Transcription Factors on Nanog promoter region of −1Kb to +0.5Kb.

Gene Name
Hoxb8, Hoxc9, Foxc1, Zic1, Hoxb9, Nr2f2, Maf, Meis1, Hoxb2, Foxc2, Pax3, Hoxa1, Osr1, Foxa1, Pax6, Gata3, Nfatc1, Foxa2, Irf2, Pitx1, Rarb, Osr2, Hand2, Pbx1, Atf3, Jun, Foxg1, Cbx4, Glis3, Sp8, Mafb, Crebbp, Ets1, Nkx2-5, Hmga2, Bach2, Twist1, Hmgn3, Ascl1, Wt1, Gli3, Pou3f2, Snai1, Vezf1, Ikzf1, Tbx15, Sox1, Arid3a, Gata6, Sox17

**Table 2:** 360 mapped proteins of identified Foxc1 interactors.

Gene Name
Mcm7, Rpl13, Ddx3x, Smarcb1, Uhrf1, Jup, Ccdc47, Hltf, Baz1b, Timmdc1, Map2k7, Timm44, Stk11, Farsa, Nob1, Dnmt1, Senp3, Trim28, Ctcf, Rps9, Rbm14, Krt14, Krt16, Pole, Rps18, Rpl10, Mta1, Cad, U2af1, Notch4, Hspa8, Prpf3, Slc25a5, Nkap, Pds5b, Myh9, Rpl19, Zc3h18, Rhot1, Cdc73, Prpf8, Kpna2, Aatf, Hdac2, Ltv1, Cdk1, Arg1, Cand1, Csrp2, Ap3d1, Ncln, Polrmt, Gnb2l1, Mmtag2, Drg1, Ddx5, Luc7l3, Eftud2, Gtpbp4, Yy1, Ssr1, Riok1, Zfp346, Mccc2, Ap3b1, Rad21, Bop1, Rpl24, Prkdc, Mcm4, Krt5, Krt2, Smarcd1, Krt6a, Krt1, Prmt5, Krt8, Chd1, Cd2ap, Hsp90ab1, Msh2, Hspa9, Ercc3, Csnk2b, Tcerg1, Lmnb1, Rps14, Smc3, Hells, Cdk2, Atp5a1, Clybl, Cbx2, Fastkd2, Hspd1, Sf3b1, Wdr12, Mcm6, Parp1, Vangl2, Nat10, Actl6a, Mccc1, Nmd3, Csde1, Arhgef2, Ndufaf4, Pdlim5, Dnaja1, Tln1, Caap1, Abcf2, Atad3a, Dhx15, Rfc1, Paics, Hsd17b11, Sdad1, Ran, Brap, Rpl6, Cul1, Asns, Prss1, Agk, Ruvbl1, Bms1, Prmt3, Copb1, Uqcrc2, Ilk, Mki67, Rps4x, Flna, Irak1, Arcn1, Smarca4, Imp3, Rpl4, Rpsa, Cct2, Abcf1, Dhx9, Noc4l, Nop14, Hnrnpk, Exoc3, Gtf3c3, Rfc3, Baz1a, Pold1, Rrp12, Aifm1, Usp22, Rpl21, Dis3, Dsc1, Tubb4b, Gltscr2, Smarca5, Thoc2, Mybbp1a, Fanci, Ptplad1, Hmgxb4, Eef2, Gnl3, Zbtb7a, Sart1, Hnrnpu, Cpsf2, Surf6, Rbm25, Pycrl, Slk, Polr2a, Picalm, Vcpip1, Exoc4, Foxc1, Fasn, Dnaja3, Heatr1, Rps23, Pcbp1, Epb4.1l2, Luc7l2, Krt9, Zfp768, Ddx17, Gtf3c1, Ubqln2, Dsg2, Rpl18a, Rrp1, Trmt10c, Chd7, Mcm3, Chd4, Zc3hav1l, Stag2, S100a8, Ddx10, Gtf2h2, Ddx1, Ubap2l, Ep300, Sbno1, Irs4, Pik3r4, Swt1, Knop1, Rps15, Gcn1l1, Micu3, Cdk11b, Rad18, Wdr6, Rpl7, Nsa2, Rps7, Prdm10, Cecr5, Pip, Upf1, Zfp512, Prpf40a, Slc25a3, Dsg1a, Tuba1b, Hnrnph1, Prpf4b, Osbpl6, Eif1a, C1qbp, Rpl17, Ewsr1, Las1l, Rps25, Utp14a, Rpl35, Krt17, Thrap3, Rrp1b, Rpl29, Rpl3, Rpl32, Timm50, Tardbp, Rfc5, Rplp0, Cdkl5, Ppp1r10, Lyar, Krt77, Adnp, Smarcc1, Smarca1, Chd8, Nt5dc2, Sry, Hist1h2ag, Naca, Rpl27, Rpl7l1, Eef1g, Mta2, Csnk2a1, Brd9, Hspa5, Sptan1, Ttf1, Rbm26, Actb, Puf60, Esyt2, Nsf, Krt10, Smarce1, Rpl23, Nol9, Nop56, Acaca, Snrnp200, Rpl23a, Acacb, Rbbp4, Rps8, Rps6, Rps27a, Xpo1, Exoc2, Ufl1, Epb4.1, Fus, Rpl38, Stk33, Pogz, Rpl18, Ruvbl2, Rps16, Arrb2, Top1, Nup93, Rbm39, Pes1, Rpl36al, Wdhd1, Gnl3l, Rif1, Amot, Ppip5k2, Fip1l1, Gfpt1, Osbpl3, Cblb, Zbtb33, Nrf1, Dsc3, Rpn2, S100a9, Ncapg, Stt3a, Gapdh, Sap30bp, Dsp, Rpl13a, Lcp1, Srsf3, Sbf1, Copa, Dpm1, Rpl22, Hnrnpm, Ythdf2, Zcchc17, Tbl3, Hnrnpdl, Rpl27a, Vangl1, Rps24, Mcm5, Krt78, Rps13, Serpinh1, Eif5, Nap1l1, Epb4.1l5, Kdm3a, Baz2a, Hnrnpf, Rpl14, Ei24, Tex10, Hnrnpd, Ddx46, Tcof1, Noc2l, Rsbn1, Nop58

## Data Availability

Data are available via ProteomeXchange with identifier PXD022178.

## Supplementary Materials

The following are available online at https://www.mdpi.com/1422-0067/22/5/2255/s1.
